# Calcium dysregulation increases right ventricular outflow tract arrhythmogenesis in rabbit model of chronic kidney disease

**DOI:** 10.1111/jcmm.17052

**Published:** 2021-11-10

**Authors:** Shih‐Yu Huang, Yao‐Chang Chen, Yu‐Hsun Kao, Yen‐Yu Lu, Yung‐Kuo Lin, Satoshi Higa, Shih‐Ann Chen, Yi‐Jen Chen

**Affiliations:** ^1^ Division of Cardiac Electrophysiology Cardiovascular Center Cathay General Hospital Taipei City Taiwan; ^2^ School of Medicine Fu Jen Catholic University New Taipei City Taiwan; ^3^ Department of Biomedical Engineering National Defense Medical Center Taipei City Taiwan; ^4^ Graduate Institute of Clinical Medicine Taipei Medical University Taipei City Taiwan; ^5^ Department of Medical Education and Research Wan Fang Hospital Taipei Medical University Taipei City Taiwan; ^6^ Division of Cardiology Department of Internal Medicine Sijhih Cathay General Hospital New Taipei City Taiwan; ^7^ Division of Cardiovascular Medicine Department of Internal Medicine Wan Fang Hospital Taipei Medical University Taipei City Taiwan; ^8^ Division of Cardiology Department of Internal Medicine School of Medicine College of Medicine Taipei Medical University Taipei City Taiwan; ^9^ Cardiac Electrophysiology and Pacing Laboratory Division of Cardiovascular Medicine Makiminato Central Hospital Urasoe Japan; ^10^ Division of Cardiology Department of Medicine Heart Rhythm Center Taipei Veterans General Hospital Taipei City Taiwan; ^11^ Cardiovascular Center Taichung Veterans General Hospital Taichung City 40705 Taiwan; ^12^ Cardiovascular Research Center Wan Fang Hospital Taipei Medical University Taipei City Taiwan

**Keywords:** calcium homeostasis, chronic kidney disease, right ventricular outflow tract, ventricular tachycardia

## Abstract

Chronic kidney disease (CKD) increases the risk of arrhythmia. The right ventricular outflow tract (RVOT) is a crucial site of ventricular tachycardia (VT) origination. We hypothesize that CKD increases RVOT arrhythmogenesis through its effects on calcium dysregulation. We analysed measurements obtained using conventional microelectrodes, patch clamp, confocal microscopy, western blotting, immunohistochemical examination and lipid peroxidation for both control and CKD (induced by 150 mg/kg neomycin and 500 mg/kg cefazolin daily) rabbit RVOT tissues or cardiomyocytes. The RVOT of CKD rabbits exhibited a short action potential duration, high incidence of tachypacing (20 Hz)‐induced sustained VT, and long duration of isoproterenol and tachypacing‐induced sustained and non‐sustained VT. Tachypacing‐induced sustained and non‐sustained VT in isoproterenol‐treated CKD RVOT tissues were attenuated by KB‐R7943 and partially inhibited by KN93 and H89. The CKD RVOT myocytes had high levels of phosphorylated CaMKII and PKA, and an increased expression of tyrosine hydroxylase‐positive neural density. The CKD RVOT myocytes exhibited large levels of *I*_to_, *I*_Kr_, NCX and L‐type calcium currents, calcium leak and malondialdehyde but low sodium current, SERCA2a activity and SR calcium content. The RVOT in CKD with oxidative stress and autonomic neuron hyperactivity exhibited calcium handling abnormalities, which contributed to the induction of VT.

## 1 INTRODUCTION

Chronic kidney disease (CKD) has become a public health burden in countries worldwide due to its increasing prevalence, poor prognosis and high mortality.^1^ The incidence of sudden cardiac death, the common aetiology of mortality in CKD, is 14 times higher among patients undergoing dialysis compared with the general population.^2^, ^3^ Clinical studies have suggested that the adverse ventricular remodelling caused by CKD provides the substrate for ventricular arrhythmia (VA).^4^, ^5^ A high frequency of VA has been reported in 48% of patients undergoing dialysis, 35% of CKD patients not undergoing dialysis with CKD and 30% of recipients of an incident kidney transplant.^5^, ^6^, ^7^ In a 24‐h Holter monitoring study of 111 patients with CKD, 35% experienced VA events in which ventricular extrasystoles were predominant.^5^ Reducing the VA burden through modified ablation of the ventricular outflow tract can improve renal function in patients with VA‐related cardiomyopathy.^8^ Moreover, advanced CKD or right ventricular outflow tract (RVOT) myocytes have been reported to have calcium handling abnormalities, abnormal electrophysiological characteristics and a low induction threshold of ventricular fibrillation.^9^, ^10^ Our previous study involving experimental animals revealed that CKD significantly increased arrhythmogenesis and fibrosis in the left atrium (but not in the right atrium), which may contribute to the high prevalence of atrial fibrillation in patients with CKD.^11^ However, the mechanism underlying CKD‐induced VA remains poorly understood.

Ventricular arrhythmias, such as premature ventricular capture, idiopathic ventricular tachycardia (VT) and torsade de pointes, frequently originate from the RVOT.^12^, ^13^, ^14^ RVOT tachycardia is a highly common type of VA.^15^ The musculature sleeves of RVOT with fibrous and fatty connective tissue possess a unique arrhythmogenic substrate or triggered activity that results in VAs.^16^ In a previous study, RVOT myocytes were shown to have a large calcium current and transient outward potassium current (*I*_to_), resulting in high RVOT arrhythmogenic activity.^10^ Moreover, sympathetic hyperactivity could influence the initiation and perpetuation of RVOT tachycardia in the tachypacing‐induced VA of a canine model.^17^ Additionally, a clinical study verified an association between oxidative stress and the genesis of RVOT VA.^18^ However, whether CKD increases RVOT arrhythmogenesis through its effects on electrical and structural remodelling remains unclear. Furthermore, the distinctive cellular pathology, electrophysiological characteristics, oxidative stress, and calcium or autonomic regulation of RVOT in CKD‐induced VA have not been elucidated. Hence, this study used a rabbit model of CKD to analyse the pathophysiological and electrophysiological characteristics, oxidative stress, calcium regulatory proteins and autonomic activity in CKD RVOT tissues and myocytes.

## 2 METHODS

### 2.1 Animal preparations

This study was approved by the ethics review board of National Defense Medical Center, where the animal experiments were conducted (IACUC‐19‐036); this study complied with the *Guide for the Care and Use of Laboratory Animals* published by the US National Institutes of Health. Further details of the animal preparations with regard to euthanasia, electrocardiographic tracings, biochemistry studies and blood pressure monitor are presented in Methods 1: Appendix S1.

### 2.2 Electropharmacological experiments in RVOT tissues

**FIGURE 1:**
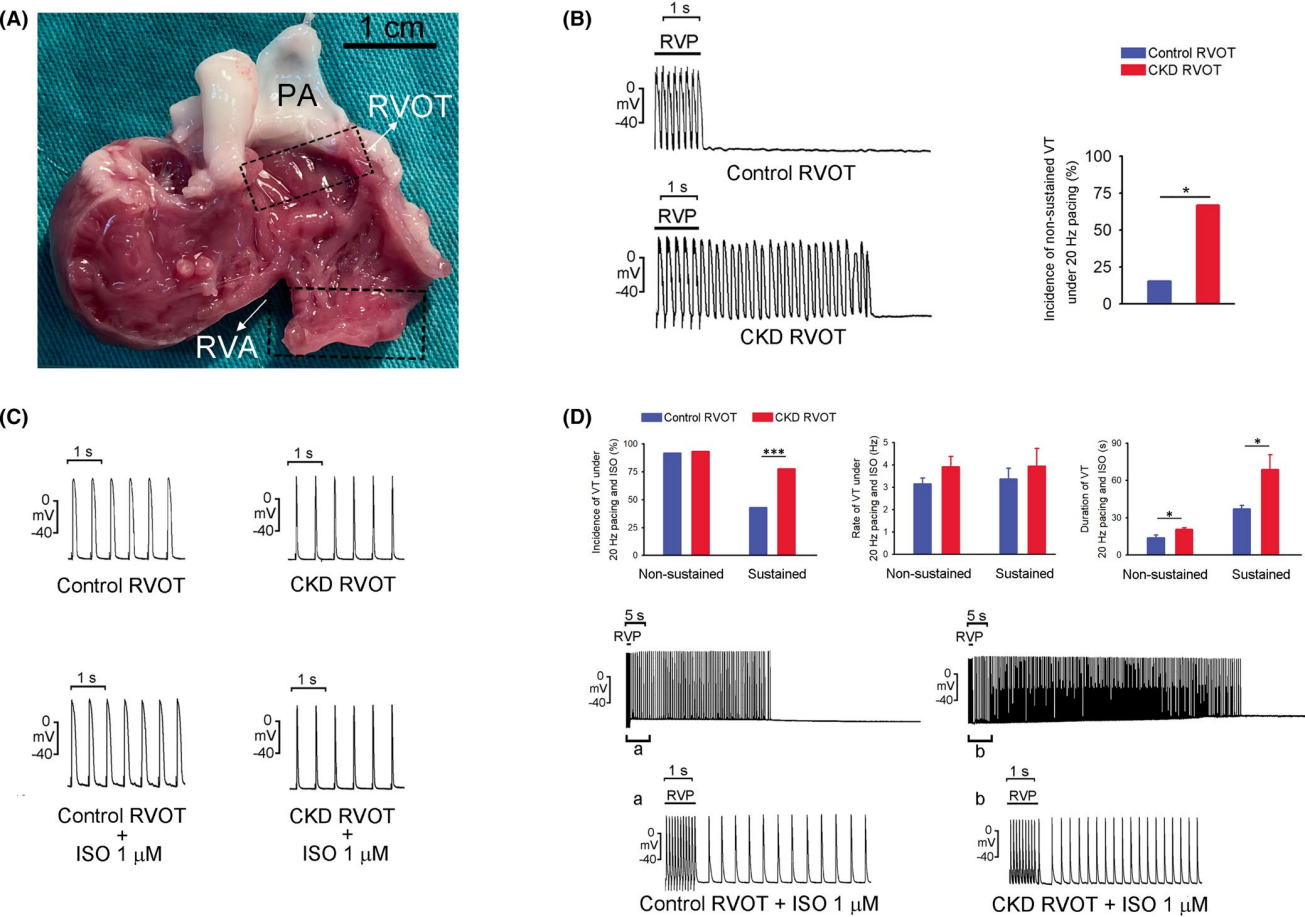
Experimental localizations of the right ventricular outflow tract (RVOT) and the effects of rapid ventricular pacing (RVP, 20 Hz) and isoproterenol (1 μM) in the RVOTs of the chronic kidney disease (CKD) and control tissues. (A) Location of the isolated RVOT tissues, superior to the supraventricular crest and ≤5 mm below the pulmonary valve. RVA and PA denote the right ventricular apex and pulmonary artery respectively. (B) Tracings and average data of RVP‐induced non‐sustained ventricular tachycardia (VT) in the CKD (*n* = 8) and control (*n* = 10) RVOT tissues. (C) Tracings of regular pacing (2 Hz) before and after the isoproterenol infusion in the CKD and control RVOT tissues. (D) Means of the incidence, beating rate and duration of RVP‐induced sustained and non‐sustained VT in the isoproterenol‐treated CKD (*n* = 17) and control (*n* = 19) RVOT tissues. The tracing indicates examples of RVP‐induced sustained VT in the isoproterenol‐treated CKD and control RVOTs. *p < 0.05, ***p < 0.005 versus control. Error bars indicate SEMs

Right ventricular outflow tract (approximately 1 cm × 1.5 cm) and RV apical tissues were isolated from all rabbits after euthanasia and prepared for analysis (Figure 1A). The RVOT was surrounded superiorly and inferiorly by the pulmonary valve and supraventricular crest, respectively, and excised at the area ≤5 mm below the pulmonary valve.^10^ The tissue preparations were bathed in 37°C Tyrode's solution containing 137 mM NaCl, 4 mM KCl, 15 mM NaHCO_3_, 0.5 mM NaH_2_PO_4_, 0.5 mM MgCl_2_, 2.7 mM CaCl_2_ and 11 mM dextrose. The tissues were superfused at a constant rate (3 ml/min) with Tyrode's solution, which was saturated with a gas mixture containing 97% O_2_ and 3% CO_2_. The transmembrane action potentials (APs) of the RVOT tissues were recorded using machine‐pulled glass capillary microelectrodes filled with 3 M KCl, and the tissue preparations were connected to a World Precision Instrument model FD223 electrometer (FL, USA).^19^ The electrical and mechanical events were displayed on a Gould 4072 oscilloscope (OH, USA) and Gould TA11 recorder simultaneously.^10^ Electrical stimuli were applied using a Grass S88 stimulator through a Grass SIU5B stimulus isolation unit. Burst firing, defined as the occurrence of an accelerated spontaneous AP with sudden onset and termination, was investigated under 2‐Hz pacing of the control and CKD RVOT tissues with or without the stimulation of isoproterenol (1 μM). Moreover, the control and CKD RVOT tissues were analysed before and after stimulation with isoproterenol (1 μM) followed by KB‐R7943 (10 μM, an inhibitor of sodium‐calcium exchanger [NCX]), KN93 (1 μM, an inhibitor of Ca^2+^/calmodulin‐dependent protein kinase II [CaMKII]), KN92 (1 μM, an inactive analogue of [KN93]) and H89 (1 μM, an inhibitor of protein kinase A [PKA]) for the observation of burst firing and effects of treatment during high‐frequency burst pacing (20 Hz) for 1 s. Sustained and non‐sustained VT were defined as tachycardias involving bursts of rapid ventricular beating lasting more and less than 30 s respectively.

### 2.3 Patch clamp experiments and measurement of intracellular calcium in isolated single cardiomyocyte preparation

Single cardiomyocytes were isolated for a patch clamp analysis that was used to measure AP duration (APD), sodium current (*I*_Na_), late sodium current (*I*_Na‐Late_), L‐type calcium current (*I*_Ca‐L_), NCX, transient outward potassium current (*I*_to_) and rapid delayed rectifier potassium current (*I*_Kr_), as described in Methods 2: Appendix S1. Intracellular Ca^2+^ concentration ([Ca^2+^]i) was recorded using a fluorometric ratio technique which was performed as shown in Methods 3: Appendix S1. The taufast and tauslow were extrapolated from the fitting of the decay phase of Ca^2+^ transient by using a bi‐exponential function.^20^

### 2.4 Sarcoplasmic reticulum Ca^2+^‐ATPase (SERCA2a) activity

The ATPase activity of the cardiac sarcoplasmic reticulum (SR) Ca^2+^ pump was measured using an enzyme‐coupled assay and SR vesicles were prepared from RVOT myocytes (see Method 4: Appendix S1).^21^, ^22^

### 2.5 Western blotting

All RVOT myocytes were centrifuged, washed with cold phosphate‐buffered saline and lysed on ice for 30 min in an RIPA buffer containing 50 mM Tris (pH 7.4), 150 mM NaCl, 1% NP40, 0.5% sodium deoxycholate, 0.1% SDS and protease inhibitor cocktails (Sigma‐Aldrich Corp.). The protein concentration was determined using a Bio‐Rad protein assay reagent (Bio‐Rad). Proteins were separated using 10% SDS‐PAGE under reducing conditions and electrophoretically transferred into an equilibrated polyvinylidene difluoride membrane (Amersham Biosciences). All blots were probed using primary antibodies against SERCA2a, NCX (Swant), CaMKII, phosphorylated CaMKII at Thr 286 (pCaMKII), ryanodine receptor (RyR) channels, PKA, connexin 43, stromal interaction molecule 1 (STIM1; BD BioScience), phosphorylated phospholamban at Ser16 (pPLB S16; GeneTex), mature collagen α1 type I (Santa Cruz Biotechnology), phosphorylated RyR at Ser2808 and Ser2814 (pRyR S2808 and pRyR S2814), phosphorylated PLB at Thr 17 (pPLB T17; Badrilla), PLB (Thermo), GADPH (MBL) and all secondary antibodies conjugated with horseradish peroxidase. All bound antibodies were detected using an enhanced chemiluminescence detection system and analysed using AlphaEaseFC software. All targeted bands were normalized to GADPH to verify the presence of equal protein loading.

### 2.6 Histological and immunohistochemistry study

Masson's trichrome stain was used to identify collagen fibres in the RVOT tissue preparations. Six images of CKD RVOTs were analysed and averaged using Image‐Pro Plus software to quantify the spectral density relative to the control RVOTs.^11^

All samples were fixed in 20% formalin prior to immunohistochemistry staining as described previously.^17^ Primary antibodies were then incubated overnight at 4°C. Antibodies against tyrosine hydroxylase (TH) and choline acetyltransferase (ChAT) were used to stain sympathetic and parasympathetic nerves respectively. The slides were washed in Tris‐buffered saline after incubation, and the appropriate secondary antibody was placed on sections for 30 min. The sections were then washed in Tris‐buffered saline again before the appropriate chromogen was added to each specimen. The specimens were dehydrated in alcohol, mounted and then examined under light microscopy. In addition, the nerve bundle was verified using positive staining of the neurofilament‐protein antibody. The TH and ChAT‐positive portions within each sample were localized and measured in their cross‐sectional areas.

### 2.7 Measurement of intracellular oxidative stress

To detect lipid peroxidation, the level of malondialdehyde (MDA) in the rabbit RVOT myocytes was assessed using an enzyme‐linked immunosorbent assay kit (ab118970, Abcam) according to the manufacturer's guidelines and a colorimetric‐fluorimetric method. The RVOT myocytes were not paced and they had a similar time between the isolation and carrying out before the MDA assay.

### 2.8 Statistical analysis

All continuous parameters are expressed in terms of the mean ± SEM. An unpaired *t* test was used to compare differences in serum, physiological and electrocardiographic characteristics of the control and CKD rabbits. Statistical significance between the control and CKD RVOT tissues and myocytes were determined using an unpaired *t* test. The results of comparisons between different regions and before versus after drug administration were analysed using a two‐way repeated measure ANOVA. The nominal data were compared using a chi‐square test with Yates’ correction or Fisher's exact test. A *p* value of <0.05 indicated statistical significance.

## 3 RESULTS

### 3.1 Electropharmacological studies in control and CKD RVOTs

The CKD rabbits had higher levels of high‐sensitivity C‐reactive protein (hs‐CRP), serum phosphate and total iron‐binding capacity but lower levels of serum calcium, iron and ferritin than the control rabbits (Table 1). As shown in Figure 1B, the CKD RVOT exhibited a higher incidence of rapid pacing (20 Hz)‐induced non‐sustained VT than the control RVOT. The electrocardiograms of the CKD rabbits indicated more prolonged RR, QT and QTc intervals, and longer QRS complex duration relative to those of the controls (Table 1).

**TABLE 1:** Serum, physiological and electrocardiographic characteristics of the control and CKD rabbits

	Control (*n* = 39)	CKD (*n* = 41)	*p* Value
BUN (mg/dl)	18.0 ± 1.4	88.6 ± 11.0	<0.001
Creatine (mmol/L)	0.097 ± 0.009	0.566 ± 0.080	<0.001
Haemoglobin (g/dl)	12.5 ± 0.3	10.8 ± 0.4	0.005
Haematocrit (%)	40.3 ± 0.5	35.3 ± 1.1	0.001
Sodium (mmol/dl)	140.6 ± 11.0	141.5 ± 1.2	0.66
Potassium (mmol/dl)	4.6 ± 0.4	4.2 ± 0.3	0.43
Calcium (mg/dl)	12.2 ± 0.05	9.8 ± 0.5	0.009
Phosphate (mg/dl)	5.4 ± 0.3	9.5 ± 1.5	0.03
Iron (µg/dl)	172.1 ± 17.8	94.5 ± 17.7	0.006
TIBC (mg/dl)	264.8 ± 10.4	318.5 ± 21.4	0.04
Ferritin (ng/ml)	0.28 ± 0.03	0.21 ± 0.02	0.04
Hs‐CRP (mg/dl)	0.002 ± 0.001	0.007 ± 0.001	0.003
SBP (mmHg)	106.1 ± 3.8	110.1 ± 3.5	0.45
DBP (mmHg)	71.0 ± 0.9	74.3 ± 2.5	0.35
RR interval (ms)	202.9 ± 8.6	243.1 ± 6.0	0.001
PR interval (ms)	70.1 ± 2.0	77.5 ± 2.4	0.06
QT interval (ms)	138.9 ± 2.6	163.7 ± 4.3	0.001
QTc interval (ms)	309.3 ± 2.2	331.1 ± 5.8	0.02
QRS duration (ms)	32.8 ± 2.4	43.9 ± 2.1	0.004

Abbreviations: BUN, blood urea nitrogen; DBP/SBP, diastolic/systolic blood pressure; Hs‐CRP, high‐sensitivity C‐reactive protein; TIBC, total iron‐binding capacity.

As shown in Figure 1C, isoproterenol (1.0 μM) could not induce VT in the CKD and control RVOTs with 2‐Hz pacing. Compared with isoproterenol (1.0 μM)‐treated control RVOTs, isoproterenol (1.0 μM)‐treated CKD RVOT exhibited a higher incidence but a similar beating rate of tachypacing‐induced sustained VT (Figure 1D). The isoproterenol‐treated CKD and control RVOTs exhibited similar incidence and beating rate of tachypacing (20 Hz)‐induced non‐sustained VT. The isoproterenol‐treated CKD RVOTs had longer durations of both tachypacing‐induced non‐sustained and sustained VT than the isoproterenol‐treated control RVOTs. Moreover, the incidence of tachypacing (20 Hz)‐induced sustained VT was higher in the isoproterenol‐treated CKD RVOTs than that in the control RVOTs.

**FIGURE 2:**
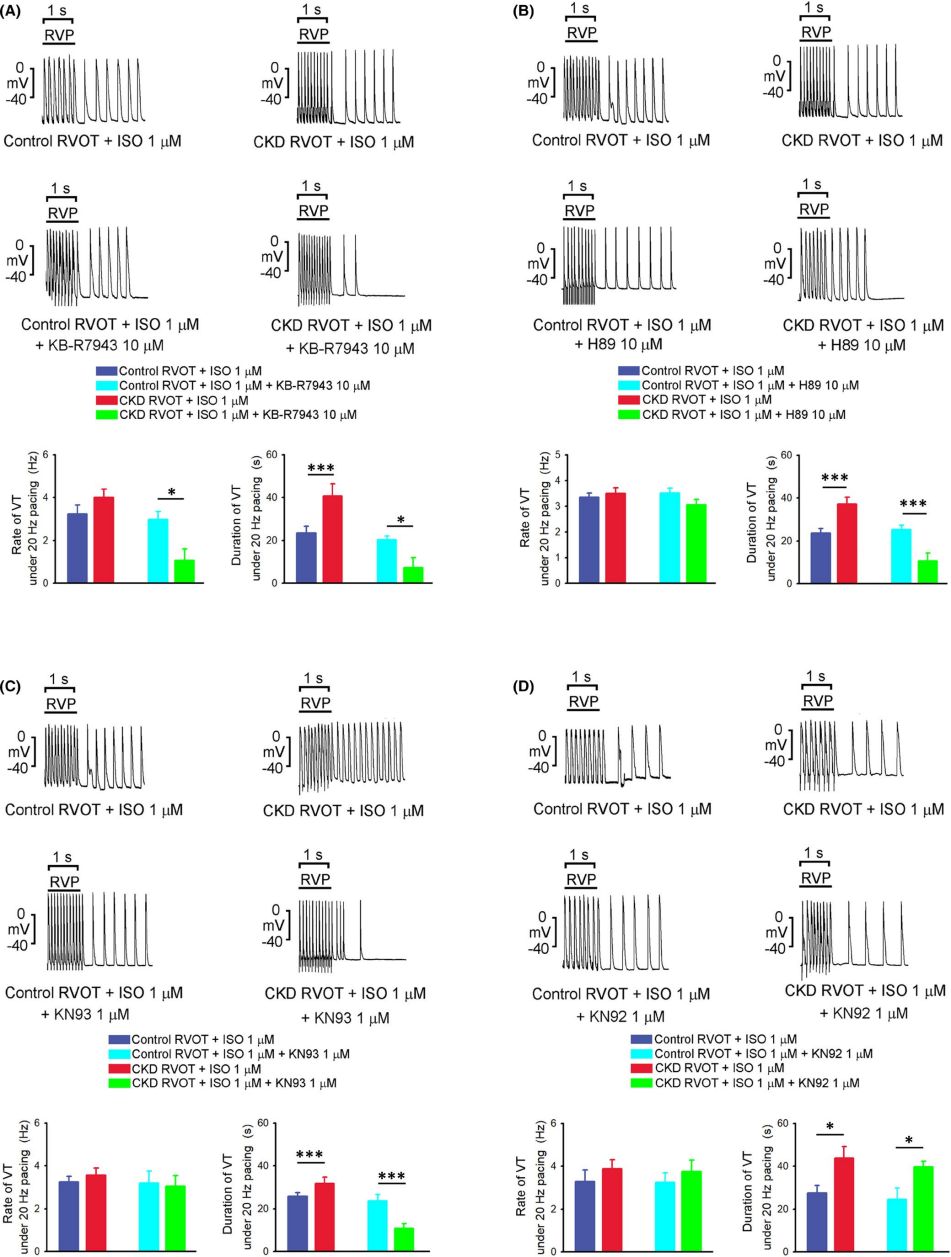
Effects of KB‐R7943 (10 μM), H89 (10 μM), KN93 (1 μM) and KN92 (1 μM) on RVP (20 Hz)‐induced VT (sustained and non‐sustained) in the isoproterenol (1 μM)‐treated CKD and control RVOT tissues. (A) Upper panel shows the tracings of RVP‐induced VT in the isoproterenol‐treated CKD (*n* = 9) and control (*n* = 10) RVOT tissues before and after KB‐R7943 (10 μM). Lower panel indicates that KB‐R7943 (10 μM) yielded a lower rate and shorter duration of RVP‐induced VT in the isoproterenol‐treated CKD RVOTs compared with those in the control RVOTs. (B) Upper panel indicates the tracings of RVP‐induced VT in the isoproterenol‐treated CKD (*n* = 6) and control (*n* = 7) RVOT tissues before and after H89 (10 μM). Lower panel indicates that H89 (10 μM) yielded a shorter duration of RVP‐induced VT in the isoproterenol‐treated CKD RVOTs compared with that in the control RVOTs. (C) Upper panel indicates the tracings of RVP‐induced VT in the isoproterenol‐treated CKD and control RVOT tissues before and after KN93 (1 μM) and KN92 (1 μM). Lower panel indicates that KN93 (1 μM) resulted in a shorter duration of RVP‐induced VT in the isoproterenol‐treated CKD RVOT tissues compared with that in the control RVOT (both *n* = 8) tissues. (D) Upper panel presents the tracings of RVP‐induced VT in the isoproterenol‐treated CKD and control RVOT tissues before and after KN92 (1 μM). However, KN92 (1 μM) did not yield a lower beating rate or shorter duration of RVP‐induced VT in the isoproterenol‐treated CKD compared with the control RVOT tissues (both *n* = 4). *p < 0.05, ***p < 0.005 versus control. Error bars indicate SEMs

As shown in Figure 2A, KB‐R7943 (10.0 μM) reduced the beating rate and duration of tachypacing (20 Hz)‐induced VT in the isoproterenol (1.0 μM)‐treated CKD RVOTs, but not in the isoproterenol (1.0 μM)‐treated control RVOTs. Moreover, during the KB‐R7943 treatment, the incidence of tachypacing‐induced VT was lower (25% vs. 88.9%, *p* < 0.05) in the isoproterenol (1.0 μM)‐treated CKD RVOTs (*n* = 8) than in the isoproterenol (1.0 μM)‐treated control RVOTs (*n* = 9). Furthermore, H89 (10.0 μM) and KN93 (1.0 μM) yielded a greater reduction in the duration (but not beating rate) of tachypacing‐induced VT in the isoproterenol‐treated CKD RVOT than in the isoproterenol (1.0 μM)‐treated control RVOTs (Figure 2B,C). However, H89 did not significantly affect the incidence of tachypacing‐induced VT in the isoproterenol‐treated CKD (83.3%, *n* = 7) or control (85.7%, *n* = 6) RVOTs. Similarly, KN93 did not significantly affect the incidence of tachypacing‐induced VT in the isoproterenol‐treated CKD (62.5%, *n* = 8) and control (75%, *n* = 8) RVOTs. KN92 (1.0 μM) did not reduce the incidence (100% in both groups, each *n* = 5), beating rate or duration of tachypacing‐induced VT in the isoproterenol‐treated CKD and control RVOTs (Figure 2D).

### 3.2 Ionic currents in control and CKD RVOTs

**FIGURE 3:**
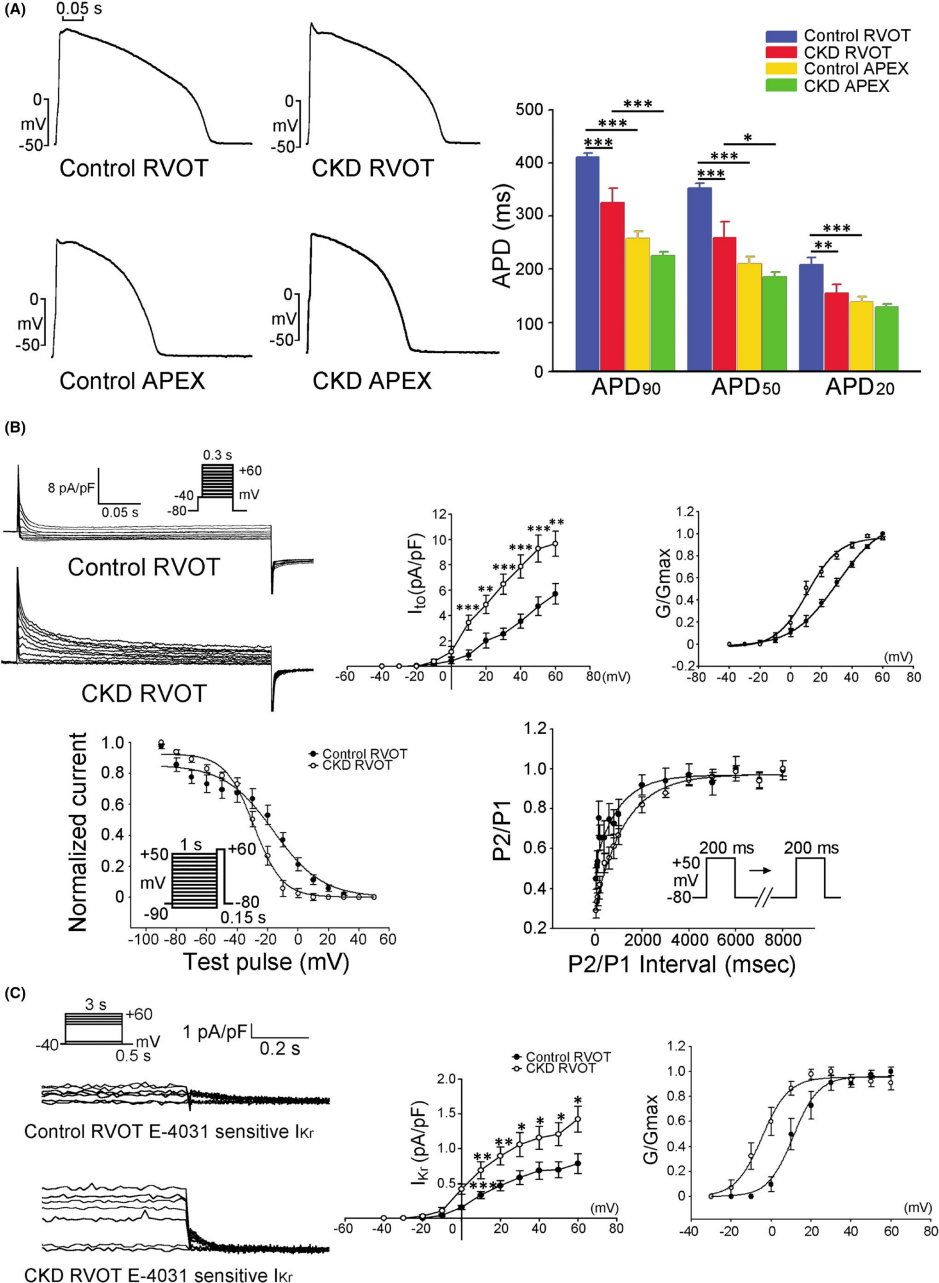
Action potential (AP) morphology in the RVOT and apical myocytes from the CKD and control rabbits and transient outward potassium current (*I*_to_) and rapid delayed rectifier potassium current (E‐4031‐sensitive repolarization current, *I*_kr_) in the CKD and control RVOT myocytes. (A) Examples and average data of the APs in the RVOT and apical myocytes from the CKD (both *n* = 8, from three hearts) and control (*n* = 10, from four and three hearts respectively) RVOTs. *p < 0.05, **p < 0.01, ***p < 0.005 versus the control or CKD RVOTs. Error bars indicate SEMs. (B) Upper panels show the examples of current traces, *I*–*V* relationship and average conductance‐voltage relationship of *I*_to_ in the CKD (*n* = 8, from three hearts) and control (*n* = 10, from four hearts) RVOT myocytes. Lower panels display the voltage dependence of inactivation and the recovery kinetics of *I*_to_ from the CKD (*n* = 8, from three hearts) and control (*n* = 10, from three hearts) RVOT myocytes. (C) Examples of the current traces, *I*–*V* relationship and average conductance‐voltage relationship of *I*_Kr_ in the CKD (*n* = 9, from three hearts) and control (*n* = 9, from three hearts) RVOT myocytes. *p < 0.05, **p < 0.01, ***p < 0.005 versus control. Error bars indicate SEMs

**FIGURE 4:**
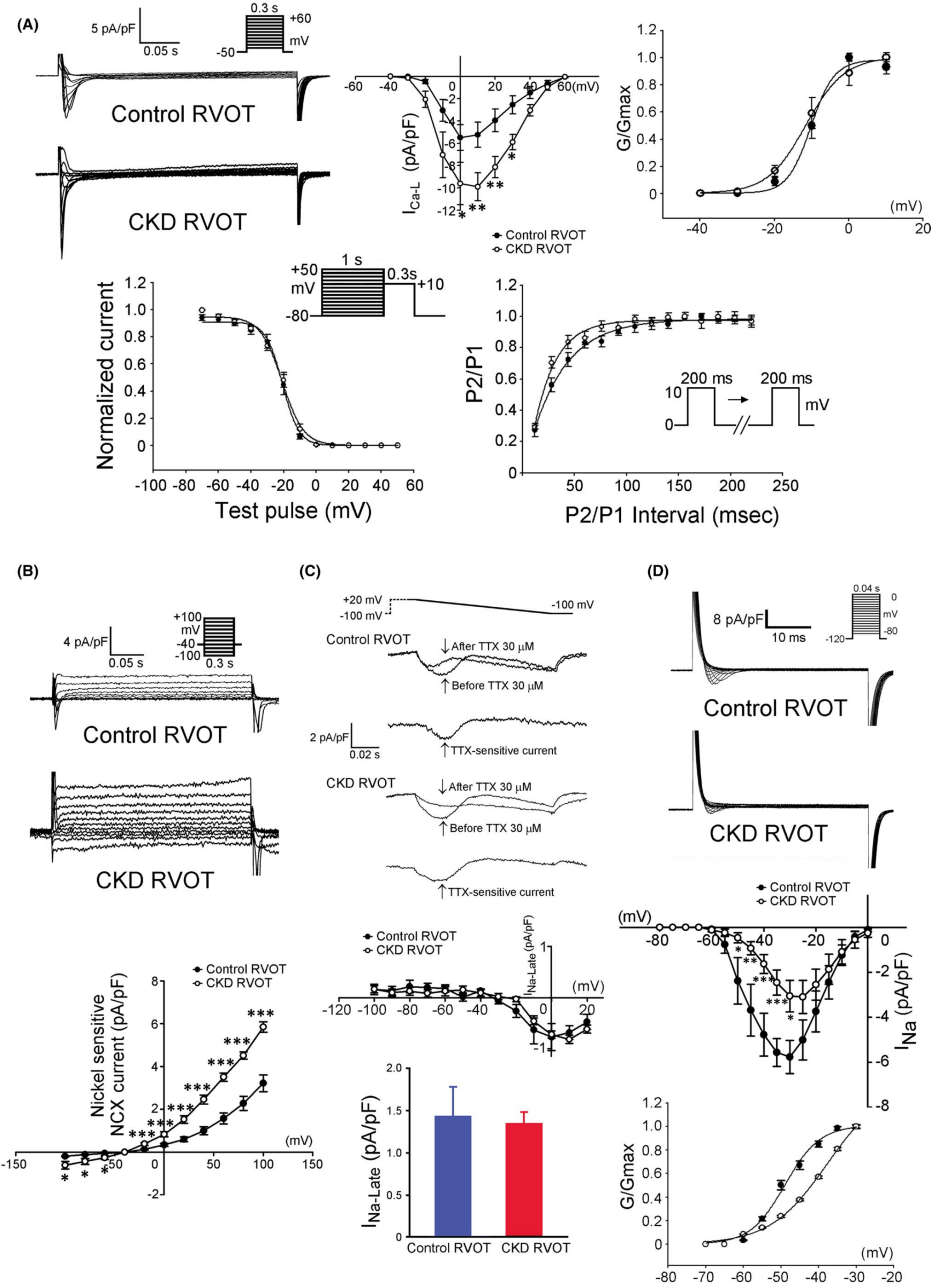
L‐type calcium (*I*_Ca‐L_), sodium‐calcium exchanger (NCX), late sodium (*I*_Na‐Late_) and sodium (*I*_Na_) currents in the CKD and control RVOT myocytes. (A) Upper panels present examples of the current traces, *I*–*V* relationship and average conductance‐voltage relationship of *I*_Ca‐L_ in the CKD (*n* = 10, from four hearts) and control (*n* = 10, from three hearts) RVOT myocytes. Lower panels present the voltage dependence of inactivation and the recovery kinetics of *I*_Ca‐L_ from CKD (*n* = 10, from three hearts) and control (*n* = 10, from three hearts) RVOT myocytes. (B) Examples of the current traces and *I*–*V* relationship of NCX in the CKD and control RVOT myocytes (both *n* = 9, from four hearts). (C) Examples of the current traces, *I*–*V* relationship and average data of *I*_Na‐Late_ in the CKD and control RVOT myocytes (both *n* = 10, from four hearts). (D) Examples of the current traces, *I*–*V* relationship, and average conductance‐voltage relationship of *I*_Na_ in the CKD and control RVOT myocytes (both *n* = 8, from three hearts). *p < 0.05, **p < 0.01, ***p < 0.005 versus control. Error bars indicate SEMs

As shown in Figure 3A, the CKD RVOT had a shorter APD at 20% repolarization (APD_20_), APD_50_ and APD_90_ than the control RVOTs, but the APDs of the CKD and control RV apical myocytes did not differ significantly. The CKD and control RV apical myocytes had shorter APD than the CKD and control RVOT myocytes respectively. These findings indicate that the effects of CKD differ between ventricular regions. The CKD and control RVOTs had similar resting membrane potentials and AP amplitudes (−73.5 ± 1.5 vs. −77.8 ± 1.7 mV and 121.7 ± 3.7 vs. 125.1 ± 3.4 mV, respectively, both *p* > 0.05). The CKD RVOTs had larger *I*_to_ and *I*_Kr_ than the control RVOTs (Figure 3B,C). The *V*_1/2_ of the activation voltage dependency of *I*_to_ and *I*_Kr_ in the CKD RVOTs was negatively shifted (21.1 ± 2.0 vs. 32.3 ± 2.1 mV, *p* < 0.05; 10.5 ± 3.2 vs. 22.5 ± 3.4 mV, *p* < 0.05 respectively) compared with that in the control. The inactivation voltage dependency of *I*_to_ currents in the CKD RVOTs was negatively shifted compared with that in control RVOTs (−30.1 ± 2.4 vs. −19.1 ± 3.3 mV, *p* < 0.05). However, CKD and control RVOTs had similar time constants of recovery from inactivation of *I*_to_ currents (1.3 ± 0.3 vs. 1.4 ± 0.5 s, *p* > 0.05, Figure 3B). The CKD RVOTs had a larger *I*_Ca‐L_ current density but a similar *V*_1/2_ of the activation voltage dependency of the *I*_Ca‐L_ current (−9.1 ± 2.7 vs. −11.2 ± 1.6 mV, *p* > 0.05) compared with the control RVOTs (Figure 4A). The CKD and control RVOTs had similar inactivation voltage dependency (−21.4 ± 1.5 vs. −22.4 ± 1.7 mV, *p* > 0.05) and time constants of recovery from inactivation of *I*_Ca‐L_ currents (36.2 ± 0.4 vs. 38.6 ± 5.2 ms, *p* > 0.05). The CKD RVOTs had larger NCX currents than the control RVOT (Figure 4B). However, the CKD and control RVOTs exhibited similar *I*_Na‐Late_ densities (Figure 4C). Additionally, the CKD RVOTs had lower *I*_Na_ currents than the control RVOTs as shown in Figure 4D. The *V*_1/2_ of the activation voltage dependency of *I*_Na_ in the CKD RVOTs was positively shifted compared with that in the control RVOTs (−42.6 ± 2.3 vs. −46.3 ± 3.2 mV, *p *< 0.05).

### 3.3 Calcium homeostasis in control and CKD RVOTs

**FIGURE 5:**
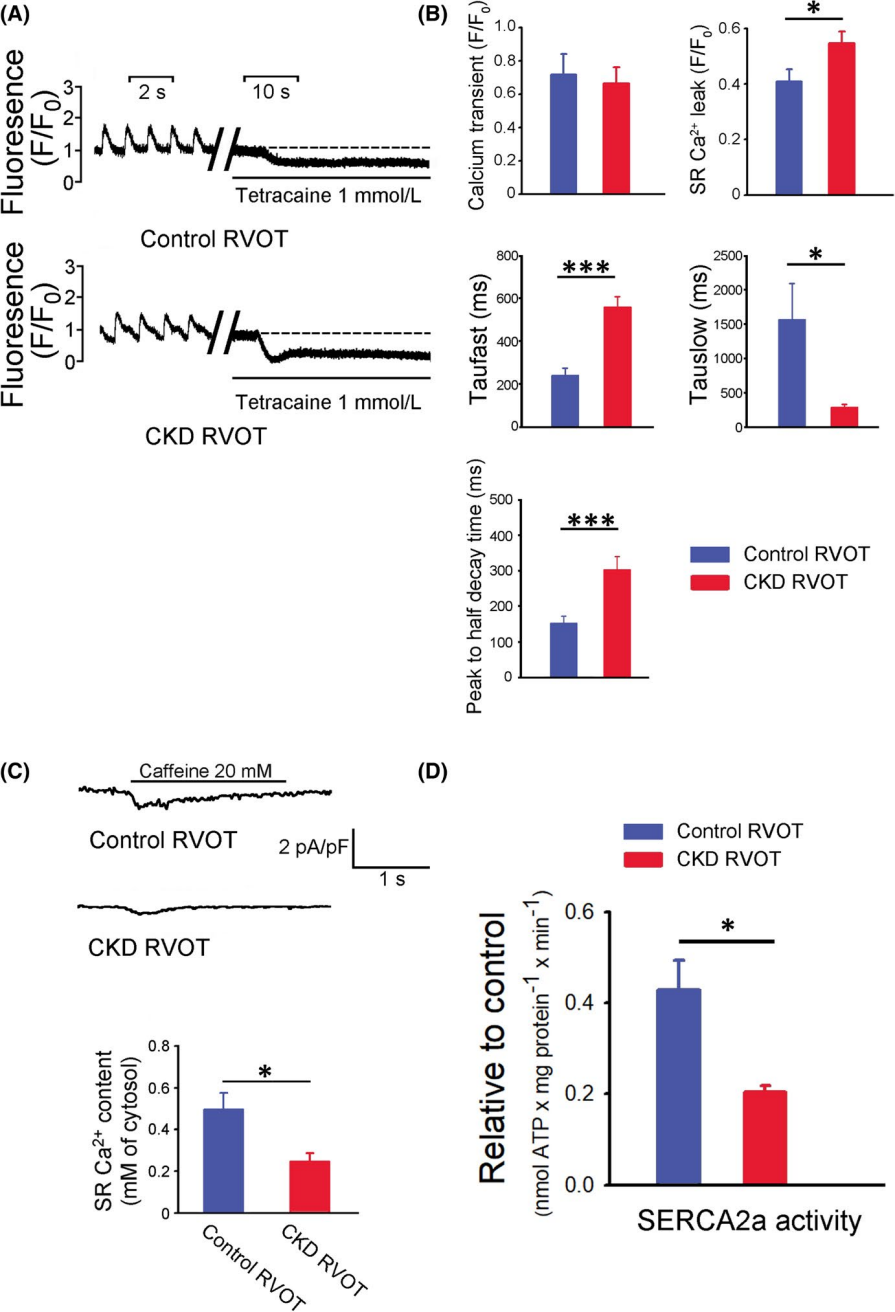
Intracellular calcium homeostasis, sarcoplasmic reticulum (SR) calcium stores and SR Ca^2+^‐ATPase (SERCA2a) activity in the CKD and control RVOT myocytes. (A) Examples of tracings of Ca^2+^ transient and SR Ca^2+^ leak in the CKD (*n* = 16, from eight hearts) and control (*n* = 16, from eight hearts) RVOT myocytes. (B) Means of Ca^2+^ transient, SR Ca^2+^ leak, taufast and tauslow of Ca^2+^ transient decay and peak to half decay time of Ca^2+^ transient in the CKD and control RVOT myocytes. (C) Examples of tracings and average data of SR Ca^2+^ content in the CKD (*n* = 8, from four hearts) and control (*n* = 11, from five hearts) RVOT myocytes. (D) Means of SERCA2a activity in the CKD and control RVOT myocytes (both *n* = 6, from three hearts). *p < 0.05, ***p < 0.005 versus control. Error bars indicate SEMs

The CKD RVOTs exhibited a larger SR Ca^2+^ leak and longer peak to half decay time of Ca^2+^ transient than the control RVOT (Figure 5A,B), but the Ca^2+^ transient was similar in the CKD and control RVOTs. Moreover, we found that the CKD RVOTs exhibited a longer taufast but a shorter tauslow than the control RVOTs (Figure 5B). The CKD RVOTs exhibited a lower SR Ca^2+^ content and SERCA2a activity than the control RVOTs (Figure 5C,D).

### 3.4 Calcium regulatory proteins, fibrosis, oxidative stress and sympathetic activity in control and CKD RVOT

**FIGURE 6:**
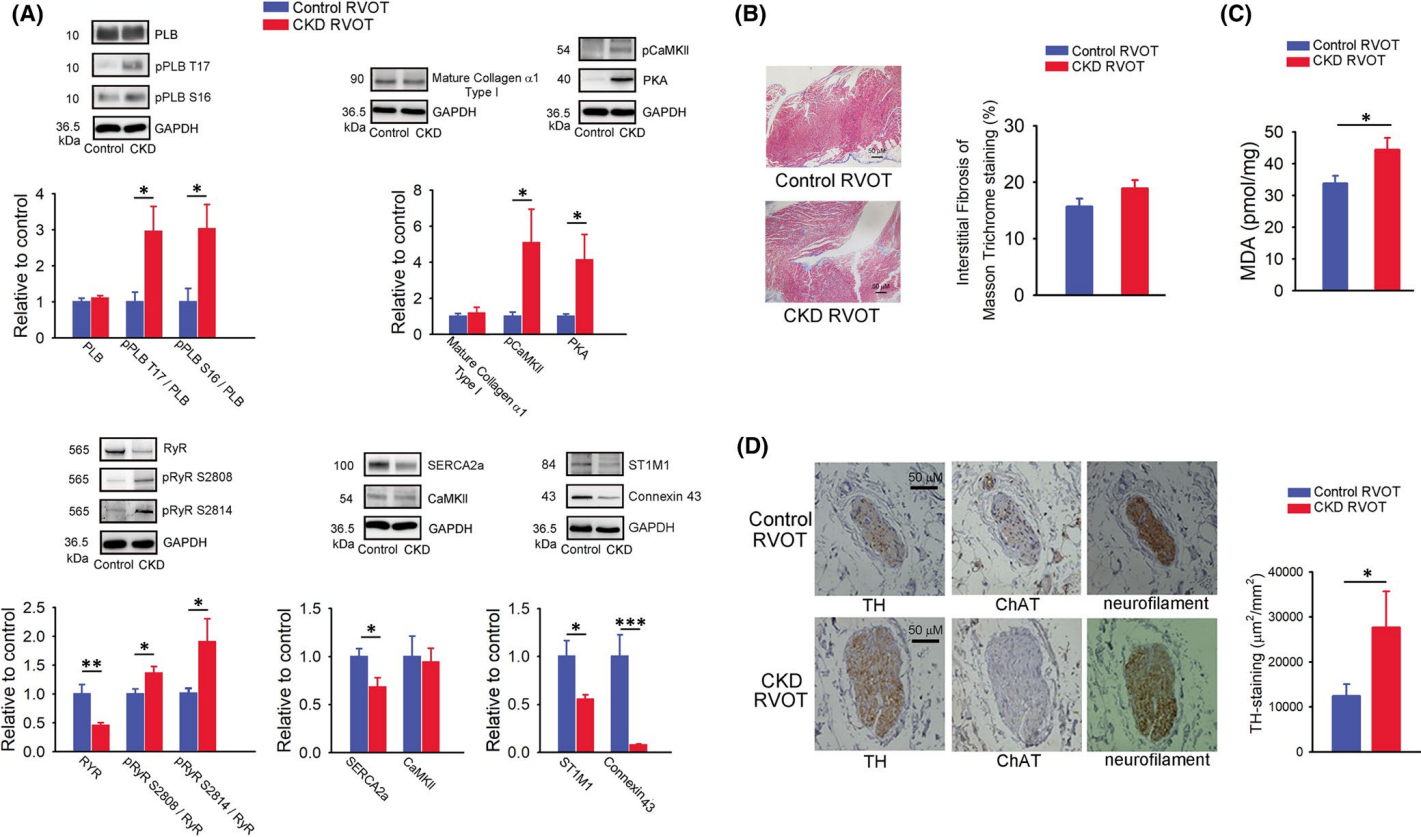
Calcium regulatory protein, histological fibrosis, malondialdehyde (MDA) and autonomic neuron activity in the CKD and control RVOTs. (A) Representative immunoblot findings and mean values of levels of phospholamban (PLB), phosphorylated PLB at threonine 17 (pPLB T17), phosphorylated PLB at serine 16 (PLB‐Ser16), ryanodine receptor channels (RyR), phosphorylated RyR at serine 2808 (pRyR S2808), phosphorylated RyR at serine 2814 (pRyR S2814), mature collagen α1 Type I, phosphorylated Ca^2+^/calmodulin‐dependent protein kinase II (pCaMKII) at Thr 286, protein kinase A (PKA), SERCA2a, CaMKII, connexin 43 and stromal interaction molecule 1 (STIM1) from the CKD and control RVOT myocytes (both *n* = 8, from four hearts). (B) Image and mean values from Masson's trichrome staining in the CKD and control RVOT tissues (both *n* = 5). (C) Mean of MDA from the CKD and control RVOT myocytes (both *n* = 6, from three hearts). (D) Image and mean values of immunohistochemistry staining for tyrosine hydroxylase (TH) and choline acetyltransferase (ChAT) in the cardiac ganglia at the CKD and control RVOT tissues (both *n* = 8). Staining of neurofilament verified the location of nerve component. *p < 0.05, **p < 0.01, ***p < 0.005 versus control. Error bars indicate SEMs

The CKD RVOT exhibited higher expression levels of pCaMKII, ratios of pPLB S16 to PLB, pPLB T17 to PLB, pRyR S2808 to RyR and pRyR S2814 to RyR, and PKA relative to the control RVOT myocytes (Figure 6A). However, the CKD RVOTs exhibited lower expression level of RyR, connexin 43 and STIM1 than the control RVOTs. The control and CKD RVOTs exhibited similar expression levels of PLB, collagen and CaMKII and similar fibrotic changes (Figure 6B). In addition, the CKD RVOTs had a higher level of MDA than the control RVOTs (Figure 6C). However, the CKD RVOTs had a higher expression level of TH as sympathetic activity relative to the control RVOTs. In contrast, the control and CKD RVOTs exhibited similar expression levels of ChAT as parasympathetic activity (Figure 6D).

## 4 DISCUSSION

This study demonstrated that CKD can alter the electrophysiological properties and induce arrhythmogenesis in an RVOT. Our previous studies have shown that the RVOT has distinctive electrical characteristics and a shorter APD than the left ventricular (LV) OT (LVOT), but a longer APD than the RV apex.^10^, ^23^ The present study suggests that CKD RVOTs exhibits a shorter APD and a higher incidence of non‐sustained VT induced by 20‐Hz rapid pacing relative to healthy RVOTs.^24^ However, a longer QT interval, determined by the sum of millions of individual ventricular repolarizations from the left (majorly) and right ventricle myocytes, was observed in the CKD rabbits than in the controls. The inconsistent effects of CKD on QT interval and RVOT APD also suggest that CKD may exert electrophysiological effects that differ between ventricular regions.^23^ Similarly, previous studies have demonstrated that a prolonged QT interval, which may be caused by electrolyte imbalance, heart failure or autonomic dysfunction, is a marker of increased cardiovascular event in patients with CKD.^25^, ^26^ Therefore, the QT interval in our CKD model may also have been prolonged by hypocalcaemia or abnormal autonomic neuron activity.

The present study observed a prolonged RR interval in CKD (as demonstrated in our previous CKD model) that may have been induced by the fibrotic sinus node and decreased funny channel expression.^11^ Furthermore, the long QRS duration observed in our CKD rabbits may have originated from a small *I*_Na_ and gap junction uncoupling caused by the changing density and channel gating of *I*_Na_ and the reduced expression of connexin 43, respectively, contributing to VA.^27^, ^28^ Previous studies in humans have demonstrated that the QRS widening is common in patients with CKD or on haemodialysis and is associated with an increased risk of mortality.^29^, ^30^, ^31^ In addition, the isoproterenol‐treated CKD RVOT tissues exhibited a higher occurrence of sustained VT induced by 20‐Hz rapid pacing compared with the control RVOT tissues. Longer durations of tachypacing‐induced non‐sustained and sustained VT were also observed in the isoproterenol‐treated CKD RVOTs. An earlier study demonstrated that post‐tachypacing may induce VA due to enhanced trigger activity from Ca^2+^ dysregulation in the RVOT.^32^ Our previous studies have proved that CKD changes calcium homeostasis in atrial or pulmonary vein cardiomyocytes.^11^, ^33^ However, further study might be required to determine calcium load by using optical mapping to identify potential mechanisms of VA related to intracellular calcium dynamics.

In the present study, short APD in the CKD RVOT myocytes may have been caused by large *I*_to_ and *I*_Kr_, which commonly facilitate the repolarization of APs. Furthermore, CKD altered the activation conductance of *I*_to_ and *I*_Kr_ in the RVOT myocytes, suggesting that the modulation of channel gating contributed to changes in the current density. A large *I*_to_ in the CKD RVOT myocytes can be consistent with the prominent spike‐and‐dome morphology of the APs required for phase 2 re‐entry.^34^ Compared with the control RVOT myocytes, the CKD RVOT myocytes had a more negative shift in the inactivation voltage dependence of *I*_to_. This indicates that the kinetic property of *I*_to_ produced a rapid repolarization of APs, contributing to its shorter APD. Moreover, this study investigates the effects of CKD on the APD of the RVOT and RV apex and the findings suggest that CKD have a discrepant effect in different RV regions. APD is the balance of inward and outward currents. Although an increase in *I*_Ca‐L_ and NCX current in CKD RVOT myocytes may counterbalance the effects of increased *I*_to_, and *I*_Kr_ on APD, a shorter APD in CKD RVOT myocytes suggests that outward potassium current may have a greater influence on APD.^35^ However, it would be useful to model RVOT AP by computer simulation to understand whether these changes are suitable to justify a shorter APD in CKD RVOT myocytes. In contrast, LV‐free wall myocytes had been shown to have a prolonged APD in CKD, which was caused by the downregulations of cardiac potassium currents.^9^ The increased regional differences of APD in CKD may contribute to an enhanced APD dispersion and facilitate the genesis of the re‐entry circuits that result in VA.^36^, ^37^

In this study, KB‐R7943, a NCX blocker, inhibited the occurrence, beating rate, and duration of tachypacing‐induced VT in the isoproterenol‐treated CKD RVOT tissues, but KN93 and H89 only shortened its duration. Although KB‐R7943 and H89 at high concentrations may exert non‐specific ionic effects (e.g. *I*_Kr_), their anti‐arrhythmic activity arose mainly from effects on NCX or CaMKII since they did not significantly change AP morphology in the RVOT myocytes (data not shown).^38^, ^39^ Although the effects of PKA and CaMKII phosphorylation or dephosphorylation on ionic currents were not evaluated in isolated single RVOT myocytes in this study, these findings suggest that increased *I*_Ca‐L_ and reverse mode of NCX give rise to calcium influx through different pathways, which could be caused by CaMKII and PKA related to oxidative stress.^33^, ^40^, ^41^, ^42^ Previous studies have demonstrated that the oxidative stress is associated with RVOT VAs and CKD‐related arrhythmogenesis, which are induced by abnormal Ca^2+^ homeostasis.^18^, ^33^ In the present study, compared with the control RVOT myocytes, the CKD RVOT myocytes exhibited similar voltage‐dependent activation and inactivation of *I*_Ca‐L_. The larger *I*_Ca‐L_ in the CKD RVOT myocytes indicates that CKD may increase the current density of *I*_Ca‐L_ in RVOTs in the absence of any modulation of ionic current conductance. Accordingly, CKD can increase calcium entry and promote calcium loading, which may cause calcium dysregulation and CaMKII signalling pathway activation.^24^ The CKD RVOT tissues also exhibited increased levels of NCX, an essential regulator of calcium homeostasis during excitation‐contraction coupling.^23^, ^43^ In heart failure, NCX is upregulated and creates conditions that facilitate arrhythmia formation through delayed afterdepolarization in a spontaneous calcium wave that elevates intracellular calcium levels during the diastolic phase.^24^, ^44^ A similar effect may be observed in CKD, which involves the suppression of arrhythmogenesis by an inhibitor of NCX, as demonstrated in this study and a previous one.^11^, ^44^ The present findings suggest that calcium homeostasis plays a key role in the RV arrhythmogenicity of CKD through the modification of abnormal calcium regulatory currents.

The signalling pathway of PKA and CaMKII and their effects on the SERCA2a pump and RyR enhance the susceptibility to cardiac arrhythmia through increased intracellular calcium load and leak respectively.^45^ The present study revealed that the CKD RVOT tissues exhibited high expression levels of pCaMKII and PKA, leading to calcium handling abnormalities. There was a decrease in SERCA2a with a long peak to half decay time of the Ca^2+^ transient under increased phosphorylation of PLB and RyR. CKD RVOT myocytes also had decreased contribution of the taufast to Ca^2+^ transient decay, suggesting a reduced SERCA2a function.^20^ In contrast, CKD RVOT myocytes exhibited an increase in NCX function during the slow decay time of the Ca^2+^ transient, which was consistent with their enhanced NCX currents that possibly originated through CaMKII‐dependent PLB phosphorylation.^46^ The CKD RVOT myocytes also manifested an increased level of oxidative stress, which may have promoted kinase activity and CaMKII expression to cause calcium dysregulation.^47^ Additionally, the protein expression of SERCA2a and SERCA2a activity may have been inhibited by the generation of oxidative stress as shown in the CKD RVOT tissues and myocytes and pressure overload‐induced heart failure cardiomyocyte as described in a previous study.^48^ Although the activity of RyR and SR Ca^2+^ leak were stimulated through the post‐translational modification of CaMKII and PKA contributing to the phosphorylation of RyR, the reduced amount of RyR in the CKD RVOT tissues may have compensated for the decreased SR content and increased intracellular calcium content.^49^, ^50^ In the present study, the expression of STIM1, located in the SR and acting as a calcium sensor that oligomerized when SR content was depleted, was reduced in the CKD RVOT myocytes. This suggests that the CKD RVOT may fail to alter SR Ca^2+^ content and regulate calcium influx resulting in the enhancement of intracellular calcium content. Moreover, the increasing calcium leak in the CKD RVOT myocytes may lead to the activation of NCX and genesis of triggered activity. In addition, the SR Ca^2+^ leak may be underestimated owing to decreased SR content in the CKD model despite its dependence on the SR Ca^2+^ load. Therefore, various downstream signalling pathways for calcium homeostasis indicated the critical role of the ventricular arrhythmogenesis of the RVOT in CKD.

The proposed mechanism underlying CKD‐related VA may involve the structural and electrophysiological remodelling of the heart, as evident in LV hypertrophy, autonomic neuron hyperactivity, cardiac fibrosis and electrolyte shifts.^4^, ^17^ The hypersympathetic innervation in the CKD RVOT suggests that the RVOT may play a role in VA induction in CKD through enhanced sympathetic activity. However, an in vivo evaluation of animal sympathetic stimulation was not undertaken in this study and the role of autonomic activity in the CKD RVOT model remains to be elucidated. Furthermore, cardiac fibrosis disrupts the myocardial structure and alters the electrophysiological properties in ventricular regions. This slows down the conduction velocity and can trigger re‐entrant VA through the heterogeneous zone of conduction and repolarization.^51^ In addition, the onset of VA can be stimulated by increased alternations of the Ca^2+^ transient and this phenomenon has been demonstrated in the CKD rats.^3^ Moreover, increased NCX currents and *I*_Ca‐L_ in our CKD RVOT myocytes during repolarization allowed a calcium extrusion and entry to compensate for the reduction of SR Ca^2+^ content induced by increased SR Ca^2+^ leak and decreased SERCA2a activity, leading to a similar amplitude of Ca^2+^ transients in the CKD and the controls.^52^ These mechanisms of cardiac arrhythmia have also been demonstrated in tissue and cellular studies in a CKD animal model.^9^, ^11^

Our CKD model, induced by antibiotics, did not present the ventricular dysfunction or structural remodelling (hypertrophy) commonly noted in patients with CKD or nephrectomy‐induced CKD animals.^9^, ^11^ This means that our model may not be useful for a study of the arrhythmogenicity of CKD RVOT tissues or myocytes in other clinical CKD conditions. Moreover, the duration of CKD can be related to the severity of renal disorientation, which is associated with VA occurrence.^3^ VA may be more complicated if the CKD durations have been longer in an anaemia model.^3^ The effects of CKD in other ventricular regions were not investigated in this study. A previous study demonstrated that heart failure modulates the electrophysiological characteristics of RVOT and LVOT myocytes differently, and heart failure prolongs the APD and increases NCX in RVOT myocytes but not in LVOT myocytes.^23^ These findings also suggest that CKD’s effect differs between ventricular regions; hence, our findings may not be applicable to other ventricular regions such as the LV. Dysregulated sodium/potassium and potassium currents as *I*_Ks_ have been demonstrated to play an important role in ventricular arrhythmogenesis in heart failure.^10^, ^23^, ^53^ In the present study, the rabbits with antibiotics‐induced CKD did not exhibit ventricular dysfunction, and the dysregulation of the potassium currents may not play a major role in the pathogenesis of pacing‐induced VA.^32^, ^54^ Therefore, this study focused on calcium homeostasis to investigate the mechanism underlying RVOT arrhythmogenesis in CKD. Additionally, increased phosphorylated CaMKII could enhance the *I*_Na‐Late_ current in the RV and LV in mice studies.^55^, ^56^ However, a previous study revealed that RVOT may feature a decreased thickness of the mid‐myocardium resulting in a regional density of *I*_Na‐late_ current that differs from that in the RV apex.^57^ Similar to a previous study, our study indicated that the CKD and control RVOT myocytes had similar *I*_Na‐Late_ current densities.^10^ In addition, this study demonstrated that CKD also changed the function of *I*_Na_ currents in RVOT myocytes. Accordingly, we speculate that *I*_Na‐Late_ in the RVOT was less affected by phosphorylated CaMKII in our CKD rabbits due to regional differences and by the combined effects of CKD on sodium currents and phosphorylated CaMKII. Consequently, further studies can identify possible interactions between these mechanisms underlying CKD RVOT‐induced arrhythmia in different models and other ventricular regions, along with their durations.

## 5 CONCLUSIONS

The RVOTs in the CKD rabbits exhibited higher oxidative stress and autonomic hyperactivity. The CKD RVOTs exhibited distinctive electrophysiological characteristics and calcium homeostasis as shortened APD and enhanced calcium leak through the modification of potassium and calcium currents and calcium regulatory proteins. The CKD‐induced RVOT remodeling may trigger a high ventricular arrhythmogenic activity.

## CONFLICT OF INTEREST

None.

## AUTHOR CONTRIBUTIONS

**Shih‐Yu Huang:** Conceptualization (equal); Data curation (equal); Funding acquisition (equal); Software (equal); Validation (equal); Writing‐original draft (equal). **Yao‐Chang Chen:** Methodology (equal); Project administration (equal); Validation (equal). **Yu‐Hsun Kao:** Methodology (equal); Writing‐original draft (equal). **Yen‐Yu Lu:** Resources (equal); Validation (equal). **Yung‐Kuo Lin:** Resources (equal); Validation (equal); Visualization (equal); Writing‐review & editing (equal). **Satoshi Higa:** Funding acquisition (equal); Visualization (equal). **Shih‐Ann Chen:** Conceptualization (equal); Investigation (equal); Supervision (equal). **Yi‐Jen Chen:** Conceptualization (equal); Investigation (equal); Project administration (equal); Supervision (equal); Writing‐review & editing (equal).

## Supporting information

Appendix S1

## Data Availability

Data available on request from the authors.
