# Supplementary material for: Calcium dysregulation increases right ventricular outflow tract arrhythmogenesis in rabbit model of chronic kidney disease
Source: J Cell Mol Med. 2021 Nov 10;25(24):11264–77. doi: 10.1111/jcmm.17052 (PMC8650029; doi:10.1111/jcmm.17052)
Supplement: Supplementary file 1 — Appendix S1 [file JCMM-25-11264-s001.docx]

**Supplementary Methods 1**

**Animal preparation**

The Male New Zealand white rabbits (weight: 2.5–3.5 kg, n = 80) received an intraperitoneal injection of vehicle or neomycin sulfate (150 mg/kg, 2 or 3 times/week) and cefazolin (500 mg/kg, 2 or 3 times/week) for 2–3 weeks as described previously.^1^ Electrocardiographic tracings of the rabbits will be recorded by connecting their 4 limbs to the cable leads of a digital Holter ECG recorder (NorthEast DR200/HE, NorthEast Monitoring Inc., MA, USA) in a restrained condition for 6 h two times a week as described previously.^1^ The detailed parameter of electrocardiograms were recorded from standard lead II limb leads via a bio-amplifier (AD Instruments, Castle Hill, Australia), connected to a polygraph recorder (ML 845 Powerlab, AD Instruments).^2^ The renal function of the rabbits in the CKD group was confirmed by assessing their serum creatinine levels (>5.0 mg/dL). The rabbits were housed under standard environmental conditions and maintained on commercial rabbit chow and tap water ad libitum. Systolic blood pressure and diastolic blood pressure of the rabbits were measured using a noninvasive blood pressure system and a high-definition oscillometry monitor (S+B medVet, New Brighton, MN). Their blood pressure was monitored at 3 and 4 weeks of age during the study. The rabbits were sacrificed at 3–4 weeks of age after they had been anesthetized using an intramuscular injection of xylazine hydrochloride (12 mg/kg) and Zoletil^®^ (Tiletamine hydrochloride/ Zolazepam hydrochloride, 12.5 mg/kg) and after they had inhaled anesthesia with isoflurane (2.0%–2.5% in oxygen) from a precision vaporizer. The anesthesia dose was confirmed as adequate because the rabbits did not exhibit corneal reflexes and motor responses to pain stimuli induced with a scalpel tip. After heparin (1000 units/kg) was intravenously administered, the heart and lungs were rapidly excised following midline thoracotomy. Furthermore, biochemical tests were performed using serum samples obtained from the rabbits.

**Supplementary Methods 2**

**Patch clamp experiments in isolated single cardiomyocytes preparation**

Single cardiomyocytes were isolated for patch clamp experiments.^3,4^ In brief, after euthanasia, the hearts were excised and mounted on a Langendorff apparatus to be superfused in an antegrade manner with oxygenated normal Tyrode’s solution at 37°C, containing 137 mM NaCl, 5.4 mM KCl, 1.8 mM CaCl_2_, 0.5 mM MgCl_2_, 10 mM HEPES, and 11 mM glucose; the pH was adjusted to 7.4 by using NaOH. After the blood was cleaned off the hearts, the perfusate was replaced with an oxygenated Ca^2+^-free Tyrode’s solution containing 300 units/mL of collagenase type I (Sigma–Aldrich, St. Louis, MO, USA) and 0.25 units/mL protease type XIV (Sigma–Aldrich) for 8–12 min. The RVOT was excised and gently shaken in a 50-mL of Ca^2+^-free oxygenated Tyrode’s solution until single cardiomyocytes were obtained as described previously.^3^ The solution was then gradually replaced by normal oxygenated Tyrode’s solution. The cardiomyocytes were allowed to stabilize in the bath for at least 30 min. before the experiments. The whole-cell patch clamp experiment was performed in the isolated RVOT and RV apical myocytes by using an Axopatch 200B amplifier (Axon Instruments, Foster City, CA, USA) at 35°C ± 1°C. Borosilicate glass electrodes (o.d., 1.8 mm) with a tip resistance of 3–5 MΩ were used. Before the formation of the membrane-pipette seal, the tip potentials were zeroed in Tyrode’s solution. The ionic currents were recorded at an approximately similar period (3–5 min) after rupture or perforation to avoid decay of ion channel activity over time. A small hyperpolarizing step from a holding potential of −50 mV to a test potential of −55 mV for 80 ms was delivered at the beginning of each experiment. The area under the capacitative current curve was divided by the applied voltage step to calculate the total cell capacitance. Normally, series resistance was electronically compensated by 60%−80%. Action potentials (APs) were elicited from isolated cardiomyocytes without spontaneous activity at a driven rate of 1 Hz for 20 beats. The diastolic membrane potential (DMP) was measured during the period between the last repolarization and the onset of the subsequent AP. The AP amplitude was obtained from DMP to the peak of AP depolarization. AP duration at 90%, 50% and 20% repolarization were respectively measured as the APD_90_, APD_50_ and APD_20_. In cells with ‘spike and dome’ shape of APs, the magnitude of phase 1 notch was measured as the membrane potential between the peak of phase 0 and the end of phase 1. Micropipettes were filled with a solution containing (in mM) KCl 20, K aspartate 110, MgCl_2_ 1, MgATP 5, HEPES 10, EGTA 0.5, LiGTP 0.1 and Na_2_ phosphocreatine 5 (pH 7.2 with KOH). Ionic currents were recorded in the voltage-clamp mode. Voltage-gated Ca^2+^ or K^+^ channel currents were plotted on the *I*–*V* curve and the curve was fitted with the modified Boltzmann equation: *I* (*V*) = [*G*_max_ × (*V* − *V*_rev_)]*/* {1 + e^[(^*^V^*^1^*^/^*^2−^*^V^*^)^*^/k^*^]^}, where *I*(*V*) is the peak current density at the command potential *V*, *G*_max_ is the maximum conductance, *V*_rev_ is the reverse potential, *V*_1/2_ is the voltage at which half-maximum IK is observed, and *k* is the slope factor.^4^

*I*_Na_ was recorded by using 40 ms pulses from a holding potential of −120 mV to the test potentials varying between −80 and 0 mV in 10 mV increments at a frequency of 3 Hz at room temperature (25±1°C). The external solution contained (in mM): NaCl 5, CsCl 133, MgCl2 2, CaCl2 1.8, nifedipine 0.002, HEPES 5 and glucose 5 (pH 7.3). Micropipettes were filled with a solution containing (in mM) CsCl 133, NaCl 5, EGTA 10, MgATP 5, TEACl 20 and HEPES 5 (pH 7.3 with CsOH).

The late sodium current (*I*_Na-Late_) included a step/ramp protocol (−100 mV stepping to +20 mV for 100 ms, then ramping back to −100 mV over 100 ms) at room temperature with an external solution containing 130 mM NaCl, 5 mM CsCl, 1 mM MgCl_2_, 1 mM CaCl_2_, 10 mM HEPES, and 10 mM glucose; pH was adjusted to 7.3 using NaOH. Micropipettes were filled with a solution containing 130 mM CsCl, 4 mM Na_2_ATP, 1 mM MgCl_2_, 10 mM EGTA, and 5 mM HEPES; pH was adjusted to 7.3 using NaOH. An equilibration period of approximately 5–10 min for dialysis was allowed to adequately clamp the cell currents. *I*_Na-Late_ was measured from the baseline to the peak of the tetrodotoxin (30 µM)-sensitive portion of the current traces obtained when the voltage was ramped back to –100 mV.^5-8^

The *I*_Ca-L_ was measured as an inward current during depolarization from a holding potential of –50 mV to test potentials ranging from –40 to +60 mV in 10 mV steps for 300 ms at a frequency of 0.1 Hz using a perforated patch clamp with amphotericin B. The micropipettes were filled with a solution containing 130 mM CsCl, 1 mM MgCl_2_, 5 mM MgATP, 10 mM HEPES, 0.1 mM NaGTP, and 5 mM Na_2_ phosphocreatine, which was titrated to a pH of 7.2 using CsOH. NaCl and KCl in the external solution were replaced with tetraethylammonium chloride and CsCl, respectively. Steady-state inactivation of *I*_Ca-L_ was evaluated using a standard protocol consisting of a 300-ms pre-pulse and a 150-ms test pulse. The peak current elicited by the test pulse was divided by the maximal current and plotted as a function of the pre-pulse voltage. Data points were fitted with a Boltzmann function. Recovery from inactivation of *I*_Ca-L_ was assessed using a two-pulse protocol with 200-ms pre- and test pulses (from –80 mV to +10 mV) separated by different time intervals. Data points were fitted with a single-exponential function.

The NCX current was elicited by depolarizing pulses between –100 and +100 mV from a holding potential of –40 mV for 300 ms at a frequency of 0.1 Hz. The amplitudes of the NCX current were measured as 10-mM nickel-sensitive currents. The external solution consisted of 140 mM NaCl, 2 mM CaCl_2_, 1 mM MgCl_2_, 5 mM HEPES, and 10 mM glucose at pH 7.4 and contained 10 μM strophanthidin, 10 μM nitrendipine, and 100 μM niflumic acid. Micropipettes were filled with a solution containing 20 mM NaCl, 110 mM CsCl, 0.4 mM MgCl_2_, 1.75 mM CaCl_2_, 20 mM TEACl, 5 mM BAPTA, 5 mM glucose, 5 mM MgATP, and 10 mM HEPES (at pH 7.25 adjusted using CsOH).

The transient outward potassium current (*I*_to_) was studied with a double-pulse protocol. A 30-ms pre-pulse from –80 to –40 mV was used to inactivate the sodium channels, followed by a 300-ms test pulse to +60 mV in 10-mV steps at a frequency of 0.1 Hz. CdCl_2_ (200 μM) was added to the bath solution to inhibit the *I*_Ca-L_. *I*_to_ was measured as the difference between the peak outward current and steady-state current. Steady-state inactivation of *I*_to_ was evaluated using a standard protocol consisting of a 1-s pre-pulse and a 0.15-s test pulse. The peak current elicited by the test pulse was divided by the maximal current and plotted as a function of pre-pulse voltage. Data points were fitted with a Boltzmann function. Recovery from inactivation of *I*_to_ was assessed using a two-pulse protocol with 200-ms pre- and test pulses (from –80 mV to +50 mV) separated by varying time intervals. Data points were fitted with a single-exponential function.

The rapid delayed rectifier potassium current (*I*_Kr_) was measured as the outward peak tail current density following a 3-s pre-pulse from a holding potential of –40 mV to voltage between –40 and +60 mV in 10-mV steps at a frequency of 0.1 Hz in the presence of E-4031 (1 μM) and CdCl_2_ (200 μM) in the Ca^2+^-free normal Tyrode’s solution. Micropipettes were filled with a solution containing 120 mM KCl, 5 mM MgCl_2_, 0.36 mM CaCl_2_, 5 mM EGTA, 5 mM HEPES 5 mM glucose, 5 mM K_2_-ATP, 5 mM Na_2_-CrP, and 0.25 mM Na-GTP (at pH 7.2 adjusted using KOH).

**Supplementary Methods 3**

**Intracellular calcium measurement**

Intracellular Ca^2+^ concentration ([Ca^2+^]i) and transient using a fluorometric ratio technique, and SERCA2a activity. ^3,9^ The control and CKD RVOT myocytes were loaded with fluorescent Ca^2+^ (10 μM) fluo-3/AM for 30 min at room temperature. Excess extracellular dye was removed by changing the bath solution, and intracellular hydrolysis of fluo-3/AM occurred at 35°C±1°C after 30 min. Fluo-3 fluorescence was excited with a 488-nm line of an argon ion laser. The emission was recorded at > 515 nm. The cells were repeatedly scanned at 2-ms intervals for a total duration of 6 s. Fluorescence imaging was performed using a laser scanning confocal microscope (Zeiss LSM 510, Carl Zeiss, Jena, Germany) and an inverted microscope (Axiovert 100). Fluorescent signals were corrected for variations in dye concentrations by normalizing the fluorescence (represented by F) against baseline fluorescence (F0) to obtain reliable information about transient intracellular Ca^2+^ (Ca^2+^i) changes from baseline values, as (F–F0)/F0, and to exclude variations in the fluorescence intensity by different volumes of injected dye. The Ca^2+^i transient, peak systolic Ca^2+^i, diastolic Ca^2+^i, and the time from peak amplitude to 50% decayed portion of the [Ca^2+^]_i_ transient were measured during a 1-Hz field-stimulation with 10-ms twice-threshold strength square-wave pulses. After achieving steady-state Ca^2+^ transients with repeated pulses from –40 to 0 mV (1 Hz for 5 s), the total amount of charge crossing the membrane sarcoplasmic reticulum (SR) Ca^2+^content (represented as Ccaff in the equation) was estimated by integrating the NCX current after rapid application of 20 mmol/L caffeine during rest with the membrane potential clamped at –40 mV to cause SR Ca^2+^ release. The SR Ca^2+^ content was measured by integrating the NCX current after rapidly adding 20 mM caffeine to the cells within 0.5 s at rest with the membrane potential clamped to –40 mV. The NCX current was measured by whole-cell patch clamp experiments. The total SR Ca^2+^ content (expressed as mmol/L of cytosol) was determined using the equation: SR Ca^2+^ content = [(1+0.12) (Ccaff/F × 1000)]/(Cm × 8.31 × 6.44), where Cm is the membrane capacitance, F is Faraday’s number, and the cell surface-to-volume ratio is 6.44 pF/pL. The SR Ca^2+^ content was estimated in some cells by rapid caffeine (20 mM) application to release SR Ca^2+^. During this procedure, the cell was imaged in the linescan mode along the longitudinal line for 15 s, which allows caffeine-evoked Ca^2+^ release to be recorded. The SR Ca^2+^ leak was measured as the tetracaine (1 mM)-reduced Ca^2+^_i_ in Na^+^ free and Ca^2+^ free solution after achieving steady-state Ca^2+^ transients with the repeated pulses (1Hz for 5 s). The time integral of the NCX currents was converted to attomolars (10^-18^ moles) of Ca^2+^ released from the SR.

**Supplementary Method 4**

**Sarcoplasmic reticulum Ca^2+^-ATPase (SERCA2a) activity**

The SERCA2a activity and sarcoplasmic reticulum (SR) vesicles were measured and prepared, respectively, from RVOT myocytes.^10,11^ SR protein (25 μg) was incubated in 250 μL buffer containing 21 mM MOPS, 100 mM KCl, 3 mM MgCl_2_, 0.06 mM EGTA, 4.9 mM NaN_3_, 1 mM glycerophosphate, 1 mM phosphoenopyruvate, 0.1 mM NADH, 8.4 units pyruvate kinase, and 12 units lactate dehydrogenase. The reactions were conducted in the presence of 1 mM ATP at 30°C (basal activity). SERCA2a activity was measured as the change in absorption at 340 nm divided by the extinction coefficient of NADH. The consumption of NADH was considered equivalent to the hydrolysis of ATP by SERCA2a with 1 μM CaCl_2_.

**References**

1. Huang SY, Chen YC, Kao YH, Hsieh MH, Chen YA, Chen WP, Lin YK, Chen SA, Chen YJ. Renal failure induces atrial arrhythmogenesis from discrepant electrophysiological remodeling and calcium regulation in pulmonary veins, sinoatrial node, and atria. *Int J Cardiol* 2016;202:846-857.

2. Lee TI, Kao YH, Chen YC, Pan NH, Lin YK, Chen YJ. Cardiac peroxisome-proliferator-activated receptor expression in hypertension co-existing with diabetes. *Clin Sci (Lond)* 2011;121:305-312.

3. Lu YY, Chung FP, Chen YC, Tsai CF, Kao YH, Chao TF, Huang JH, Chen SA, Chen YJ. Distinctive electrophysiological characteristics of right ventricular out-flow tract cardiomyocytes. *J Cell Mol Med* 2014;18:1540-1548.

4. Lu YY, Cheng CC, Tsai CF, Lin YK, Lee TI, Chen YC, Chen SA, Chen YJ. Discrepant effects of heart failure on electrophysiological property in right ventricular outflow tract and left ventricular outflow tract cardiomyocytes. *Clin Sci (Lond)* 2017;131:1317-1327.

5. Orth PM, Hesketh JC, Mak CK, Yang Y, Lin S, Beatch GN, Ezrin AM, Fedida D. RSD1235 blocks late INa and suppresses early afterdepolarizations and torsades de pointes induced by class III agents. *Cardiovasc Res* 2006;70:486-496.

6. Lin YK, Chen YC, Chen JH, Chen SA, Chen YJ. Adipocytes modulate the electrophysiology of atrial myocytes: implications in obesity-induced atrial fibrillation. *Basic Res Cardiol* 2012;107:293.

7. Noble D, Noble PJ. Late sodium current in the pathophysiology of cardiovascular disease: consequences of sodium-calcium overload. *Heart* 2006;92 Suppl 4:iv1-iv5.

8. Ton AT, Nguyen W, Sweat K, Miron Y, Hernandez E, Wong T, Geft V, Macias A, Espinoza A, Truong K, Rasoul L, Stafford A, Cotta T, Mai C, Indersmitten T, Page G, Miller PE, Ghetti A, Abi-Gerges N. Arrhythmogenic and antiarrhythmic actions of late sustained sodium current in the adult human heart. *Sci Rep* 2021;11:12014.

9. Huang SY, Chen YC, Kao YH, Hsieh MH, Lin YK, Chen SA, Chen YJ. Redox and Activation of Protein Kinase A Dysregulates Calcium Homeostasis in Pulmonary Vein Cardiomyocytes of Chronic Kidney Disease. *J Am Heart Assoc* 2017;6.

10. Munch G, Bolck B, Karczewski P, Schwinger RH. Evidence for calcineurin-mediated regulation of SERCA 2a activity in human myocardium. *J Mol Cell Cardiol* 2002;34:321-334.

11. Chu A, Fill M, Stefani E, Entman ML. Cytoplasmic Ca2+ does not inhibit the cardiac muscle sarcoplasmic reticulum ryanodine receptor Ca2+ channel, although Ca(2+)-induced Ca2+ inactivation of Ca2+ release is observed in native vesicles. *J Membr Biol* 1993;135:49-59.
